# Genomic Identification and Functional Analysis of JHAMTs in the Pond Wolf Spider, *Pardosa pseudoannulata*

**DOI:** 10.3390/ijms222111721

**Published:** 2021-10-29

**Authors:** Zhi-Ming Yang, Yong Wu, Fang-Fang Li, Zhang-Jin Zhou, Na Yu, Ze-Wen Liu

**Affiliations:** Key Laboratory of Integrated Management of Crop Diseases and Pests (Ministry of Education), College of Plant Protection, Nanjing Agricultural University, Nanjing 210095, China; 2018202054@njau.edu.cn (Z.-M.Y.); 2020102122@stu.njau.edu.cn (Y.W.); 2019102137@njau.edu.cn (F.-F.L.); 2020102124@stu.njau.edu.cn (Z.-J.Z.); yuna@njau.edu.cn (N.Y.)

**Keywords:** *Pardosa pseudoannulata*, JHAMT, phylogeny, RNA interference, gene expansion

## Abstract

Juvenile hormone (JH) plays a critical role in many physiological activities of Arthropoda. Juvenile hormone acid methyltransferase (JHAMT) is involved in the last steps of JH biosynthesis as an important rate-limiting enzyme. In recent studies, an increasing number of JHAMTs were identified in arthropods, but no JHAMT was reported in spiders. Herein, eight JHAMTs were identified in the pond wolf spider, *Pardosa pseudoannulata*, all containing the well conserved S-adenosyl-L-methionine binding motif. *JHAMT-1* and the other seven *JHAMTs* were located at chromosome 13 and chromosome 1, respectively. Multiple alignment and phylogenetic analysis showed that JHAMT-1 was grouped together with insect JHAMTs independently and shared high similarities with insect JHAMTs compared to the other seven JHAMTs. In addition, *JHAMT-1*, *JHAMT-2*, and *JHAMT-3* were highly expressed in the abdomen of spiderlings and could respond to the stimulation of exogenous farnesoic acid. Meanwhile, knockdown of these three *JHAMTs* caused the overweight and accelerated molting of spiderlings. These results demonstrated the cooperation of multi-JHAMTs in spider development and provided a new evolutionary perspective of the expansion of *JHAMT* in Arachnida.

## 1. Introduction

Juvenile hormone (JH), the sesquiterpenoid hormone, regulates development, metamorphosis, reproduction, and diapause in Arthropoda [1,2,3,4,5,6]. Juvenile hormone acid methyltransferase (JHAMT) is a highly specific rate-limiting enzyme in JH biosynthesis in insects [7]. It belongs to the family of S-adenosyl-L-methionine (SAM)-dependent methyltransferases (MTs) which transfer a methyl group from methyl donor SAM to the carboxyl group of substrates [7,8]. JHAMT catalyzes the methylation in the last steps of juvenile hormone III (JH III) biosynthesis, converting farnesoic acid (FA) to methyl farnesoate (MF) or converting JH III acid (JHA III) to JH III [1,7].

Since the first JHAMT was identified in *Bombyx mori* [7], a great number of orthologs of JHAMT have been characterized in insects, such as *Drosophila melanogaster* [9], *Tribolium castaneum* [10], *Aedes aegypti* [11], *Acyrthosiphon pisum* [12], *Apis mellifera* [13], *Leptinotarsa decemlineata* [14], *Blattella germanica* [15], and so on. Silencing *JHAMT* lowered JH titer [14], caused larval precocious metamorphosis [10], and inhibited vitellogenin synthesis [15]. Taken together, JHAMT was highly correlated to JH biosynthesis and played essential roles in metamorphosis and reproduction in insects.

Recently, with the developments of the deep sequenced genomes and transcriptomes, JHAMT orthologs were also identified in Arachnida, including mite *Tetranychus urticae* [16,17], scorpion *Mesobuthus martensii* [18], ticks *Ixodes scapularis* [19,20], *Dermacentor variabilis* [20], and *Ornithodoros turicata* [20]. However, no study related to the identification and function of JHAMT in JH biosynthesis was reported in spiders. In this study, we took advantage of the genomic and transcriptomic databases to identify and characterize JHAMTs in the pond wolf spider *Pardosa pseudoannulata*. In addition, the function of JHAMTs in spider development was demonstrated by RNA interference (RNAi).

## 2. Results

### 2.1. Characterization of JHAMTs

**Figure 1:**
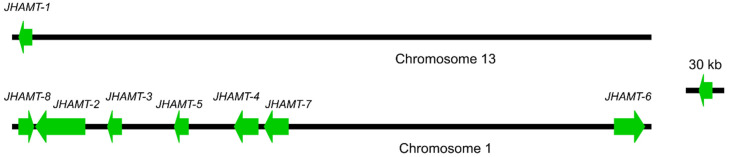
Chromosomal distribution of eight *JHAMT* genes in *P. pseudoannulata*. Only regions (black line) containing target genes were illustrated at the chromosomes. Green arrows represented genes and indicated the relative direction of genes to the chromosome.

**Figure 2:**
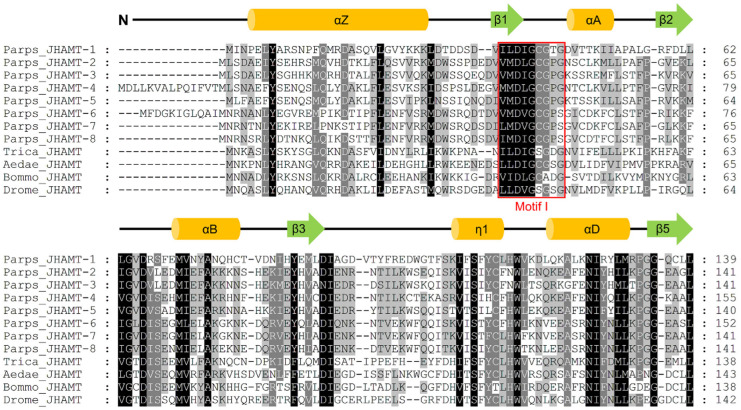

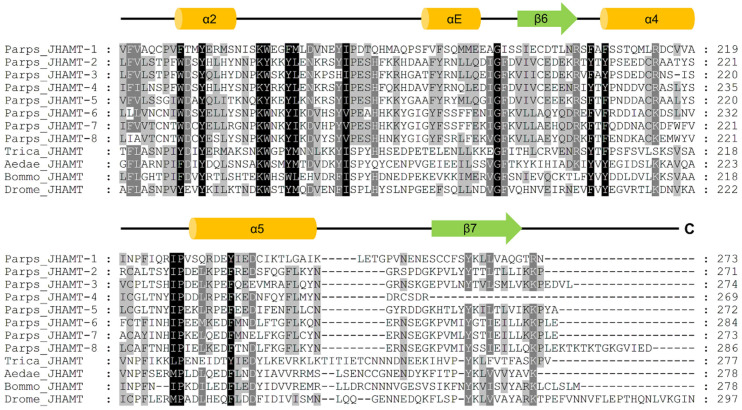
Conserved regions of JHAMTs from *P. pseudoannulata* and insects. Residues highlighted by red box were the conserved S-adenosyl-L-methionine binding motif (motif I). Secondary structural elements of JHAMTs were showed as orange cylinders (α-helices) and green arrows (β-strands) marked above the sequences. Parps, *Pardosa pseudoannulata*; Trica, *Tribolium castaneum*; Aedae, *Aedes aegypti*; Bommo, *Bombyx mori*; Drome, *Drosophila melanogaster*.

Eight full-length *JHAMTs* were identified in *P. pseudoannulata*, namely, *JHAMT-1*, -*2*, -*3*, -*4*, -*5*, -*6*, *-7*, and *-8*, and submitted to GenBank (GenBank accession number: MZ321024, MZ321025, MZ321026, MZ321027, MZ321028, MZ321029, MZ321030, and MZ321031, respectively). Their open reading frames (ORFs) were 822 base pairs (bp), 816 bp, 825 bp, 810 bp, 819 bp, 855 bp, 822 bp, and 861 bp which encoded a protein of 273 amino acids (aa), 271 aa, 274 aa, 269 aa, 272 aa, 284 aa, 273 aa, and 286 aa, respectively. The accuracy of the complete sequence was confirmed by PCR (Figure S1). Eight *JHAMTs* were located at two chromosomes with *JHAMT-1* at chromosome 13 and the rests at chromosome 1 (Figure 1). The predicted amino acid sequences of eight JHAMTs contained the well conserved SAM-binding motif (motif I) and their secondary structure incorporated the alternation of nine α-helices and six β-strands comparing with the typical core fold of SAM-MTs (Figure 2).

### 2.2. Phylogenetic Analysis

**Figure 3:**
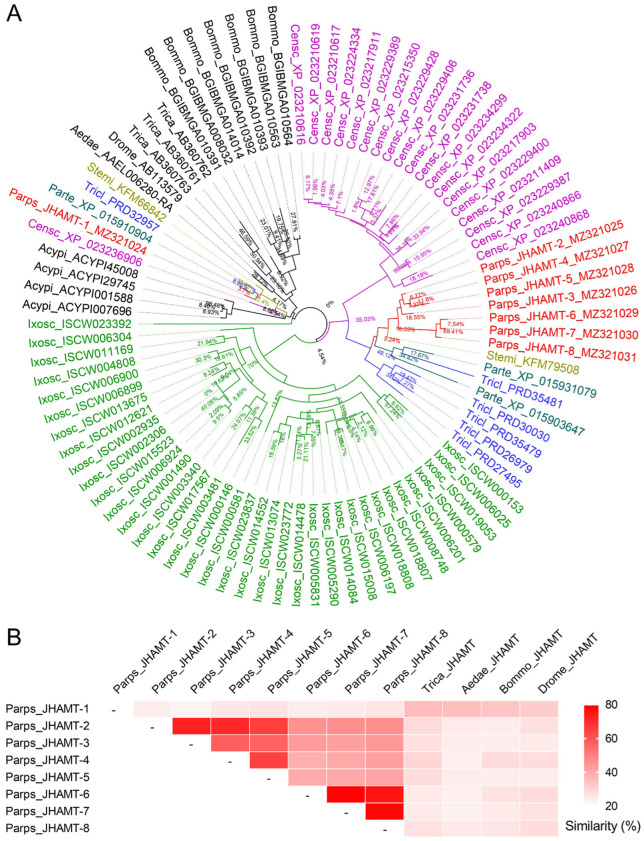
Phylogenetic analysis (**A**) and similarities (**B**) of JHAMTs from arachnids and insects. These JHAMTs were retrieved from the previous reports [9,10,11,19,21,22] or identified in the present study (Table S2). The gene ID was represented by the abbreviation of the Latin name of the species & its accession number. The phylogenetic tree was constructed by IQ-TREE and processed in Figtree software. The black JHAMTs were insects. The JHAMTs from an arachnid species were marked with the same color. The numbers at base of nodes were the branch times. The heatmap of similarities was processed in GraphPad Prism 7. Bommo, *Bombyx mori*; Aedae, *Aedes aegypti*; Trica, *Tribolium castaneum*; Drome, *Drosophila melanogaster*; Acypi, *Acyrthosiphon pisum*; Ixosc, *Ixodes scapularis*; Parps, *Pardosa pseudoannulata*; Parte, *Parasteatoda tepidariorum*; Stemi, *Stegodyphus mimosarum*; Tricl, *Trichonephila clavipes*; Censc, *Centruroides sculpturatus*.

The numbers of 3, 2, 6, 1, 1, and 20 *JHAMT* genes were identified from genomes of three spiders *P. tepidariorum*, *S. mimosarum*, and *T. clavipes*, two mites *V. destructor* and *G. occidentalis*, and a scorpion *C. sculpturatus*, respectively (Figure 3A, Table S2). It was very difficult to strict distinguish the JHAMTs between arachnids and insects according to the phylogenetic analysis of JHAMTs because one of the multi-JHAMT copies from arachnids was grouped together with those in insects and the remaining arachnid JHAMTs were grouped together (Figure 3A). Meanwhile, there were differences in similarities of amino acid sequences between eight JHAMTs from *P. pseudoannulata* and four JHAMTs from insects (Figure 3B). In *P. pseudoannulata*, JHAMT-1 shared the similarities of 24%, 23%, 26%, 27%, 24%, 25%, and 26% with JHAMT-2, -3, -4, -5, -6, -7, and -8, respectively, which were lower than that of four JHAMTs from insects with over 32% similarities. Among the other seven JHAMTs from *P. pseudoannulata*, the similarities were 57–72% among JHAMT-2, -3, -4, and -5, and 75–79% among JHAMT-6, -7, and -8, and 39–46% between these two groups (Figure 3B).

### 2.3. Spatiotemporal Expression Profile

**Figure 4:**
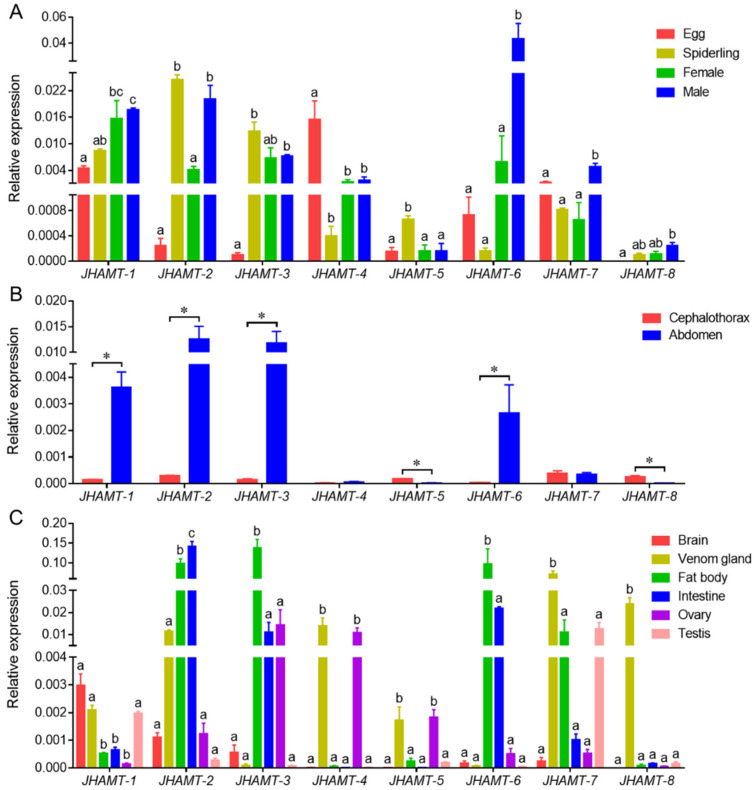
Spatiotemporal expression profiles of eight *JHAMT* genes in four developmental stages (egg, the second-instar spiderling, adult female, and adult male) (**A**), two parts (cephalothorax and abdomen) from the second-instar spiderlings (**B**), and six tissues (brain, venom gland, fat body, intestine, ovary, and testes) from adult females and males (**C**). Different lower-case letters (a, b, or c) indicated the significant difference of gene expression in four developmental samples and in six tissular samples at *p* < 0.05. *, *p* < 0.05.

There were different expression profiles of eight *JHAMTs* in four developmental stages, egg, the second-instar spiderling, adult female and adult male (Figure 4A). *JHAMT-1* was expressed higher in males and lower in eggs. *JHAMT-2* was significantly expressed in spiderlings and males rather than eggs and females. *JHAMT-3* and *JHAMT-4* showed the opposite expression patterns with *JHAMT-3* highly expressed in spiderlings and adults, while *JHAMT-4* highly expressed in eggs. Both *JHAMT-5* and *JHAMT-8* showed low expression levels in the four samples. Both *JHAMT-6* and *JHAMT-7* were significantly expressed in males, especially *JHAMT-6* (Figure 4A). The spiderlings were separated into two parts, cephalothorax and abdomen (Figure 4B). *JHAMT-1*, *JHAMT-2*, *JHAMT-3*, and *JHAMT-6* were significantly expressed in abdomen. Both *JHAMT-5* and *JHAMT-8* were significantly expressed in cephalothorax. There was no expressional difference of *JHAMT-4* and *JHAMT-7* between the two parts (Figure 4B). Spatial expressions of eight *JHAMTs* showed the specific patterns in six tissues, brain, venom gland, fat body, intestine, ovary, and testes (Figure 4C). *JHAMT-1* was highly expressed in brains, venom glands, and testes. *JHAMT-2* was significantly expressed in fat bodies and intestines. Both *JHAMT-3* and *JHAMT-6* showed significant expressions in fat bodies. Both *JHAMT-4* and *JHAMT-5* showed remarkable expressions in venom glands and ovaries, and traces in the rest tissues. Both *JHAMT-7* and *JHAMT-8* showed notable expressions in venom glands, especially *JHAMT-8* (Figure 4C).

### 2.4. Effect of Farnesoic Acid Administration

**Figure 5:**
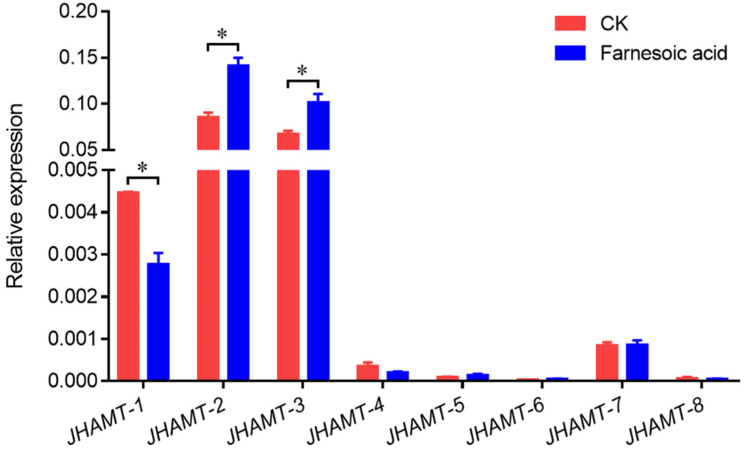
Relative expressions of eight *JHAMT* genes in farnesoic acid-applied the second-instar spiderlings. CK, control group containing 200 time-diluted ethanol. *, *p* < 0.05.

To determine the function of eight JHAMTs in JH synthesis, the changes of *JHAMT* transcriptional level were detected in spiderlings treated by FA. The transcriptional level of *JHAMT-1* was significantly downregulated after FA treatment, but both *JHAMT-2* and *JHAMT-3* were significantly stimulated (Figure 5). In addition, the other 5 *JHAMTs* showed no difference between FA treatment and control group (Figure 5).

### 2.5. Effect of JHAMT Silencing

**Figure 6:**
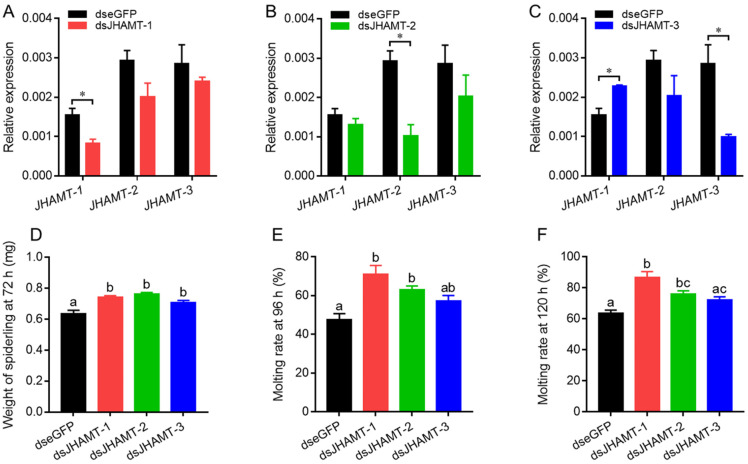
Effects of injection of dsJHAMT-1, dsJHAMT-2, and dsJHAMT-3 in the second-instar spiderlings. Relative expressions of *JHAMT-1*, *JHAMT-2*, and *JHAMT-3* in spiderlings treated by dsJHAMT-1 (**A**), dsJHAMT-2 (**B**), and dsJHAMT-3 (**C**). Weight of spiderlings at 72 h (**D**) and molting rates of spiderlings at 96 h (**E**) and 120 h (**F**) after injection of dseGFP and three dsJHAMT. Different lower-case letters (a, b, or c) indicated the significant difference of weight and molting rate of spiderlings between dseGFP and three dsJHAMT groups at *p* < 0.05. *, *p* < 0.05.

Compared with dseGFP-treated spiderlings, *JHAMT-1*, *JHAMT-2*, and *JHAMT-3* were downregulated by 47% (Figure 6A), 65% (Figure 6B), and 65% (Figure 6C) in target dsJHAMT-treated spiderlings, respectively. The expressions of the other non-target *JHAMTs* were not changed in dsJHAMT treatments except for the increase of *JHAMT-1* expression in dsJHAMT-3-treated spiderlings (Figure 6C). The spiderlings of both control and treatment groups were weighted at 72 h after injection. The average weight of dseGFP, dsJHAMT-1, dsJHAMT-2, and dsJHAMT-3-treated spiderlings were 0.63 mg, 0.74 mg, 0.76 mg, and 0.71 mg, respectively (Figure 6D). The spiderlings in the three dsJHAMT treatment groups were weighted heavier than dseGFP group. In addition, the molting rates of spiderlings were 48%, 71%, 63%, and 57% at 96 h in dseGFP, dsJHAMT-1, dsJHAMT-2, and dsJHAMT-3 groups, respectively (Figure 6E), and at 120 h, they were 64%, 87%, 76%, and 72%, respectively (Figure 6F). The molting rates of the three dsJHAMT treatment groups had statistical difference with dseGFP group except for dsJHAMT-3 group in both counts.

## 3. Discussion

In the present study, eight *JHAMTs* were identified in the whole genome of *P. pseudoannulata*. It was the first detailed characterization of JHMAT in spiders. Similarly, multi-copies of *JHAMT* were also found in several arachnids, including three spiders *P. tepidariorum*, *S. mimosarum*, and *T. clavipes*, and a scorpion *C. sculpturatus*. Moreover, the numbers of 3 and 44 *JHAMT* genes have been reported in the genomic works of scorpion *M. martensii* [18] and tick *I. scapularis* [19], respectively. However, the same with *T. urticae* [16,17], only one *JHAMT* presented in two mite species, *V. destructor* and *G. occidentalis*. Therefore, there was the striking expansion of *JHAMT* in spiders, scorpions, and ticks, but not in mites. The duplication of *JHAMT* also occurred in insects, including *T. castaneum* [10], *A. pisum* [12], and *B. mori* [21], although only one *JHAMT* in most insect species. Interesting results from the phylogenetic tree showed that there was always a JHAMT in arachnids that was independent of the other copies and grouped together with that of insects. Further, JHAMT-1 from *P. pseudoannulata* showed high similarities of amino acid sequences with insect JHAMTs than the other seven JHAMTs. In the chromosomal distribution of eight *JHAMT* genes, *JHAMT-1* was located at chromosome 13 and the remaining *JHAMTs* were located together at chromosome 1. From the above results, we speculated that an ancestral *JHAMT* gene was presented in both Arachnida and Insecta, and the new *JHAMTs* were developed and duplicated in arachnids after the separation of insects. In the future, more evidence related genomic analyses of arachnid species are needed to confirm this hypothesis.

JHAMT, as the key rate-limiting enzyme in regulation of JH titer, is involved in the last steps of the active JH product biosynthesis pathway in insects by transferring the methyl group of SAM to the carboxyl group of FA or JHA III [7]. Differing from corpora allata as the main biosynthetic site of JH III in insects [23], these JH biosynthesis-related genes were highly expressed in abdomen of *P. pseudoannulata* [24]. It indicated that *JHAMT-1*, *JHAMT-2*, *JHAMT-3*, and *JAHMT-6* were involved in JH biosynthesis in *P. pseudoannulata* due to their specific expressions in abdomen. Just as predicted, the gene expressions of *JHAMT-1*, *JHAMT-2*, and *JAHMT-3* were significantly changed by exogenous FA application, but not *JHAMT-6*. *JHAMT-2* and *JHAMT-3* were stimulated by FA, but opposite in *JHAMT-1*. It might be that there was a cooperative mechanism between the JHAMTs to respond to the changes of FA. In addition, some JHAMTs might have functional differentiation, such as *JHAMT-7* and *JHAMT-8* involved in toxin production in the spider because of their high expressions in venom glands.

The development of spiderlings was affected by *JHAMT* silencing. *JHAMT* downregulation could increase the weight of spiderlings. Meanwhile, the accelerated molting was embodied in the higher molting rate of dsJHAMT treatments than that of control group at both 96 h and 120 h. However, JHAMT-3 had no effects in spiderling’s development although there was a statistical difference in the weight of spiderlings, possibly due to the functional absence of JHAMT-3, which was rescued by the increased expression of *JHAMT-1* in dsJHAMT-3-treated spiderlings. To sum up, JHAMTs regulate spider development in a coordinated way.

## 4. Materials and Methods

### 4.1. Identification and Characterization of JHAMTs

The putative JHAMTs were retrieved from the protein database predicated from the chromosome-level genome of *P. pseudoannulata* (GenBank accession number: JAGEOH000000000) using the orthologs from *B. mori* [7], *D. melanogaster* [9], *T. castaneum* [10], and *A. aegypti* [11] (GenBank accession number: BAC98835, BAC98836, BAG30999, and EAT42177, respectively) as query proteins via the command-line tool Exonerate (version 2.2, EMBL-EBI, Cambridge, UK) [25]. The obtained JHAMT sequences were confirmed by the nine transcriptomes of *P. pseudoannulata* (GenBank accession number: SRR8083389-SRR8083396, and SRR8083398, respectively) and manually corrected gap prediction and UTR to get the complete ORF by multiple alignment in Clustal X (version 2.1, University College Dublin, Dublin, Ireland) [26]. The complete ORF of *JHAMT* was confirmed by PCR. The specific primers were designed using Beacon Designer (version 7.92, PREMIER Biosoft, San Francisco, CA, USA) (Table S1) and synthesized by Genscript (Genscript, Nanjing, China). The PCR products were sequenced in Tsingke (Tsingke Biotechnology, Nanjing, China). The putative JHAMTs were also surveyed in six arachnids which had genomic database entries using the same methods, including *Parasteatoda tepidariorum*, *Stegodyphus mimosarum*, *Trichonephila clavipes*, *Varroa destructor*, *Galendromus occidentalis*, and *Centruroides sculpturatus* (downloaded from https://www.ncbi.nlm.nih.gov/genome/, the genomic database of *T. clavipes* last accessed on 18 October 2019, the rest were last accessed on 18 March 2021). Multiple alignment was performed by Clustal X [26] and illustrated in GENEDOC (version 2.7, downloaded from https://genedoc.software.informer.com/download/, accessed on 29 March 2016) [27]. The maximum likelihood phylogenetic tree was constructed by IQ-TREE (version 2.1.3, downloaded from http://www.iqtree.org/, accessed on 22 October 2021) [28] and processed in Figtree (version 1.4.3, downloaded from https://github.com/rambaut/figtree/releases, accessed on 4 October 2016). Secondary structure was derived from the consistent predictions of different tools, including JPred4 (http://www.compbio.dundee.ac.uk/jpred/, accessed on 24 April 2020) [29], PSIPRED (http://bioinf.cs.ucl.ac.uk/psipred/, accessed on 24 April 2020) [30], and PredicProtein (https://www.predictprotein.org/, accessed on 24 April 2020) [31]. The predicted topology of the fold was presented based on previous descriptions [8,11]. Gene locations at chromosome were drawn by IBS (version 1.0.3, downloaded from http://ibs.biocuckoo.org/download.php, accessed on 5 June 2018) [32].

### 4.2. Spiders

*P. pseudoannulata* adults were collected from a rice field in Nanjing (Jiangsu province, China) in May 2020 and housed in 500 mL plastic cups individually at 28 ± 1 °C and 16/8 h light/dark and fed with *Nilaparvata lugens*. The spiders were reared in laboratory conditions for at least one month before experiments began. Four developmental samples, egg, the second-instar spiderling, adult female, and adult male, were collected individually and 5–10 eggsacs or spiders were pooled as one sample. Two parts, cephalothorax and abdomen, were dissected from 10 the second-instar spiderlings. Six tissular samples, brain, venom gland, fat body, intestine, ovary, and testes, were dissected from 20 adult females and males. Each sample was carried out with three biological replicates.

### 4.3. Farnesoic Acid Treatment

FA was purchased from Echelon (Salt Lake City, UT, USA) and dissolved in absolute ethanol to get the stock solution of 10 mg/mL. The stock solution was diluted using sterilized water to get the working concentration of 0.05 mg/mL. Ethanol diluted 200 times was set as negative control. The day 1 second-instar spiderlings were starved in petri dishes (3.5 cm in diameter) individually for 12 h before bioassay. The working solution soaked in absorbent cotton was supplied to each spiderling and solution-cotton was refreshed every 24 h. The spiderlings were fed with a few *N. lugens* after exposure to working solution for 36 h. Ten spiderlings were harvested at 72 h to pool as one sample and each sample was carried out with three biological replicates.

### 4.4. RNA Interference

The gene specific primers with extended T7 RNA polymerase promoter sequence on the 5′ end were designed using Beacon Designer (Table S1) and synthesized by Genscript. The gene fragment was amplified using Phanta^®^ Max Super-Fidelity DNA Polymerase (Vazyme, Nanjing, China) and then purified using GeneJET Gel Extraction Kit (Thermo Scientific, Carlsbad, CA, USA) according to the manufacturer’s instructions. dsRNA was synthesized using T7 RiboMAX™ Express RNAi System (Promega, Madison, WI, USA) according to the manufacturer’s instructions. The integrity and quantity of dsRNA were verified by 1.5% agarose gel electrophoresis and NanoPhotometer spectrophotometer (IMPLEN, Westlake Village, CA, USA) respectively. dsRNA against enhanced green fluorescent protein (*eGFP*, GenBank accession number: KC896843) was used as the negative control. Delivery of dsRNA by injection have been described in the previous report [33]. This method was used to investigate the biological function of JHMATs in *P. pseudoannulata* in the present study. Briefly, after anaesthetization by carbon dioxide, the day 1 second-instar spiderlings were kept in an agar gel plate and microinjected with 10 nL of 50 ng dsRNA individually from the injection site of ventral abdomen. The injected-spiderlings were transferred into petri dishes individually and fed with *N. lugens*. Individuals died of mechanical injury within 12 h were removed. Injected spiderlings were divided into two groups. Group I was used for quantitative PCR (qPCR). The number of 10 spiderlings of each dsRNA treatment were harvested at 48 h to pool as one sample. Group II was used for phenotypic responses. The number of 15–20 spiderlings of each dsRNA treatment were used to record the phenotypes. The spiderlings were weighted at 72 h and the counts of molts were recorded at both 96 h and 120 h. The experiment was conducted three times independently.

### 4.5. Real-Time Quantitative PCR

Total RNA was extracted using Trizol™ reagent (Invitrogen, Carlsbad, CA, USA) according to the manufacturer’s instructions. The integrity and quantity of RNA were verified by 1.5% agarose gel electrophoresis and NanoPhotometer spectrophotometer (IMPLEN, Westlake Village, CA, USA) respectively. cDNA was synthesized using PrimeScript RT Reagent Kit (TaKaRa, Kyoto, Japan) according to the manufacturer’s instructions. Two reference genes of elongation factor-1 alpha (*EF-1α*, GenBank accession number: KJ888948) and glyceraldehyde-3-phosphatedehydrogenase (*GAPDH*, GenBank accession number: KJ888949) were selected based on previous description [33]. Primers for qPCR were designed using Beacon Designer (Table S1) and synthesized by Genscript. The specificity and efficiency of the primers were verified via melting curve and standard curve assay respectively. The components of qPCR reaction were made using TB Green Premix Ex Taq II Kit (TaKaRa, Kyoto, Japan) according to the manufacturer’s instructions and performed on QuantStudio Real-Time PCR System (Applied Biosystems, Foster City, CA, USA). Each reaction was carried out with two technical replicates.

### 4.6. Data Analysis

The relative gene expression was related to the geometric mean of two reference genes by the 2^−ΔCT^ method [34,35]. Gene expression, weight, and molting rate of spiderlings were presented as mean ± SEM from three independent biological replicates. Significant differences were analyzed with t-tests for gene expressions between cephalothorax and abdomen, FA treatments, and dsRNA treatments, and with one-way ANOVA followed by Tukey test for gene expressions in four developmental samples and six tissular samples, and weights and molting rates of spiderlings in dsRNA treatments using GraphPad Prism (version 7, GraphPad Software, San Diego, CA, USA) [36].

## Supplementary Materials

The following are available online at https://www.mdpi.com/article/10.3390/ijms222111721/s1.
